# Predictive Model for Complications in Total Hip Arthroplasty: Development of a Clinical Tool

**DOI:** 10.1055/s-0046-1824726

**Published:** 2026-10-06

**Authors:** Andrea Moreira Candido, Conrado Torres Laett, Alanna Martins Soares de Palma, Lourenço Pinto Peixoto, Sidnei Cavalcante da Silva

**Affiliations:** 1 Programa de Pós-Graduação em Ciências Aplicadas ao Sistema Musculoesquelético, Divisão de Ensino e Pesquisa (DIENP), Instituto Nacional de Traumatologia e Ortopedia (INTO), Rio de Janeiro, RJ, Brazil; 2 Laboratório de Pesquisa Neuromuscular, Divisão de Pesquisa, Instituto Nacional de Traumatologia e Ortopedia (INTO), Rio de Janeiro, RJ, Brazil; 3 Centro de Cirurgia do Quadril, Divisão de Traumatologia e Ortopedia (DITRO), Instituto Nacional de Traumatologia e Ortopedia (INTO), Rio de Janeiro, RJ, Brazil; 4 Programa de Pós-Graduação em Ciências da Reabilitação, Centro Universitário Augusto Motta (UNISUAM), Rio de Janeiro, RJ, Brazil; 5 Laboratório de Pesquisa em Fisiologia do Exercício, Divisão de Pesquisa, Instituto Nacional de Traumatologia e Ortopedia (INTO), Rio de Janeiro, RJ, Brazil

**Keywords:** arthroplasty, replacement, hip, predictive learning models, prognostic factor, risk factors, artroplastia de quadril, fatores prognósticos, fatores de risco, modelos preditivos de aprendizagem

## Abstract

**Objective:**

To develop a predictive model to estimate complications up to 30 days after total hip arthroplasty (THA), based on clinical and functional variables, aiming at the creation of a practical tool for clinical use.

**Methods:**

Prospective cohort study conducted in a public referral hospital, with patients undergoing primary and elective THA between November 2021 and April 2022. The sample included 122 patients (mean age: 56 years, 59.8% men). Clinical, sociodemographic, and functional capacity variables were evaluated and estimated by the Duke Activity Status Index (DASI). Outcomes included clinical complications, early readmission, prolonged hospitalization (> 6 days), and intensive care unit (ICU) stay for more than one day. A logistic regression model was used, with performance evaluated by the C-statistic (area under the curve, AUC), and the model was incorporated into an online tool.

**Results:**

The most prevalent comorbidities were hypertension (61%), diabetes (23%), and dyslipidemia (15%). Complications within 30 days occurred in 13.1% of patients, with surgical wound infection and anemia being the most common events. Dyslipidemia (odds ratio [OR]: 4.70; *p* = 0.010), sedentary lifestyle (OR: 4.71; *p* = 0.026), high ASA classification (OR: 3.67; *p* = 0.035), and functional capacity < 4 metabolic equivalents of task (METs) (OR: 4.08; *p* = 0.040) were significant risk factors. The tool is available at https://upneuro.shinyapps.io/atq-pred .

**Conclusion:**

Dyslipidemia, sedentary lifestyle, low functional capacity, and high ASA classification were associated with a higher risk of post-THA complications. The preoperative evaluation should include functional and cardiovascular aspects. The model showed good accuracy, and further studies are needed to validate its clinical application.

**Level II – Moderate level evidence.:**

This is a prospective cohort study without a control group aimed at identifying prognostic factors for complications after total hip arthroplasty.

## Resumo

**Objetivo:**

Desenvolver um modelo preditivo para estimar complicações até 30 dias após artroplastia total de quadril (ATQ), com base em variáveis clínicas e funcionais, visando à criação de uma ferramenta prática para uso clínico.

**Métodos:**

Estudo de coorte prospectiva realizado em hospital público de referência, com pacientes submetidos à ATQ primária e eletiva entre novembro de 2021 e abril de 2022. A amostra incluiu 122 pacientes (média de idade: 56 anos, 59,8% homens). Foram avaliadas variáveis clínicas, sociodemográficas e de capacidade funcional, estimada pelo Duke Activity Status Index (DASI). Os desfechos incluíram intercorrências clínicas, reinternação precoce, internação prolongada (> 6 dias) e permanência em UTI por mais de um dia. Utilizou-se modelo de regressão logística, com desempenho avaliado pela estatística C (AUC), incorporado a uma ferramenta online.

**Resultados:**

As comorbidades mais prevalentes foram hipertensão (61%), diabetes (23%) e dislipidemia (15%). Complicações em até 30 dias ocorreram em 13,1% dos pacientes, sendo infecção da ferida operatória e anemia os eventos mais comuns. Dislipidemia (RC: 4,70; *p* = 0,010), sedentarismo (RC: 4,71; *p* = 0,026), ASA elevado (RC: 3,67; *p* = 0,035) e capacidade funcional < 4 equivalentes metabólicos da tarefa (METs, do inglês *metabolic equivalents of task* ) (RC: 4,08; *p* = 0,040) foram fatores de risco significativos. A ferramenta está disponível em https://upneuro.shinyapps.io/atq-pred .

**Conclusão:**

Dislipidemia, sedentarismo, baixa capacidade funcional e ASA elevada associaram-se ao maior risco de complicações pós-ATQ. A avaliação pré-operatória deve incluir aspectos funcionais e cardiovasculares. O modelo mostrou boa acurácia, mas novos estudos são necessários para validar sua aplicação clínica.

**Nível II – Evidência de nível moderado.:**

Trata-se de um estudo de coorte prospectivo, sem grupo controle, voltado à identificação de fatores prognósticos para complicações em artroplastia total de quadril.

## Introduction

Total hip arthroplasty (THA) is the surgical procedure that replaces the hip joint with a prosthesis, aiming to reestablish joint function and relieve pain. It is one of the most performed and successful elective surgeries worldwide, with more than a million procedures per year 1 2 being considered by many to be the orthopedic surgery of the century due to strong clinical outcomes. 3

Despite advances in perioperative care, complications are still frequent in the postoperative period, with morbidity at 30 days after THA ranging between 5 and 20%. 4 5 6

Preoperative risk stratification allows us to identify potentially modifiable factors, optimize clinical conduct, guide intra- and postoperative strategies, and assist in resource management. 7 8 9 Additionally, predictive models contribute to shared decision-making between medical staff and patients regarding the procedure's performance. 10

Several risk indices or scores are used to predict complications in noncardiac surgeries, such as the American Society of Anesthesiology (ASA) classification, the Revised Cardiac Risk Index (RCRI), and the Multicenter Perioperative Outcomes Group (MPOG) score. 11 12 13 To improve this prediction in orthopedic surgeries, Waterman et al. 14 proposed the Cardiac Risk Index for Total Joint Arthroplasty (CRI-TJA), based on data from the US population. 15

Among the main factors associated with morbidity and mortality after THA are: age over 75 years, ASA score ≥ 3, male gender, and comorbidities, such as cardiovascular disease, systemic arterial hypertension (SAH), obesity, and cerebrovascular disease. 14 16 Although studies point to low functional capacity as a relevant risk factor for adverse events in noncardiac surgeries, 17 18 the limitations imposed by orthopedic diseases hinder its objective evaluation in THA candidates. In these cases, instruments like the Duke Activity Status Index (DASI) 19 may represent a viable alternative for assessing functional capacity in an indirect and standardized manner. According to Wijeysundera et al., 20 DASI was associated with a higher risk of complications or death within 30 days after major non-cardiac surgeries.

In the Brazilian population, the identification of risk factors and the development of a tool to predict complications after THA remain areas for exploration, which is the objective of the present study.

## Materials and Methods

Patients undergoing elective primary THA at our institution from November 16, 2021, to April 16, 2022, were invited to participate in a prospective cohort with a 30-day follow-up to evaluate adverse events. Patients older than 18 years were included, while those with cancelled surgery or who were unable to understand the questionnaires were excluded. The data obtained were used to develop a prognostic model. The study was previously approved by the institution's human research ethics committee (CAAE: 36510920.8.0000.5273), and all participants signed an informed consent form.

The patients' physical capacity was evaluated using the DASI, translated and validated into Portuguese, which was administered during an interview by the responsible researcher. 21 The questionnaire consists of 12 items with varying weights, the sum of which is used to estimate the maximum oxygen consumption per minute (VO _2max_ ) through the equation described by Hlatky et al. 22 Furthermore, VO _2max_ was expressed in metabolic equivalents of task (METs), with 1 MET: 3.5 L/min. To minimize interference with the usual hospitalization procedure, demographic data, laboratory tests, and ASA classification were obtained from the medical record. Patient data were used for preoperative risk stratification according to RCRI, CRI-TJA, and MPOG.

The patients were followed by an analysis of medical records from the start of surgery until the 30 ^th^ postoperative day. Moderate or severe complications were considered adverse events according to the Clavien-Dindo classification, as adapted by Wijeysundera et al. 23 Moderate and severe complications are, respectively, conditions that require treatment but do not cause permanent damage, and conditions that pose a potential risk of death and/or require admission to the intensive care unit.

Continuous, discrete, and dichotomous numerical data are reported as mean ± standard deviation (SD), median (interquartile range [IQR]), and absolute occurrence (percentage). The association between the recorded factors and events was evaluated using the odds ratio (OR) from univariate logistic regression; variables with *p* -values < 0.10 in the Wald test were included in a multivariate logistic model. Independent risk factors were those with *p* < 0.05 in the univariate Wald test.

The performance of the logistic regression model and risk stratification scores was evaluated by the area under the receiver operating characteristic curve (C-statistic) and compared by the DeLong method. The developed model was integrated into an online risk calculation tool for the project (UPNEURO-INTO, https://upneuro.shinyapps.io/atq-pred ). All statistical analyses were performed using Python 3.10 routines (Python Software Foundation).

## Results

In total, 122 patients participated in the study, with primary osteoarthritis (OA) being the indication in 86 cases (70.4%) and secondary OA in 36 (29.6%). Secondary causes were trauma, osteonecrosis, developmental dysplasia of the hip, and sequelae of septic arthritis. Among them, 16 (13.1%) experienced adverse events during the 30-day follow-up, with complications occurring during initial hospitalization in 10 patients (8.2%), as well as 6 readmissions (4.9%). Furthermore, two patients had multiple complications, one with anemia requiring blood transfusion and surgical wound infection, and the other with anemia requiring blood transfusion, tachyarrhythmia, and hypertensive crisis.

The complications observed alone were in three cases of anemia requiring blood transfusion, two cases of prosthesis dislocations, one case of acute respiratory infection (ARI), one case of tachyarrhythmia, and one case of exacerbation of chronic obstructive pulmonary disease (COPD). In four cases, the cause of readmission was surgical wound infection, in another it was periprosthetic infection, and in the remaining case, suspicion of deep vein thrombosis (DVT).

It was possible to identify risk factors for adverse events, presented in detail along with the sample characteristics in Table 1 .

**Table 1:** Characteristics of participants and risk factors

	Total	Affected patients	Non-affected patients	*p* -value	OR	95% CI
**Male**	60% (73)	56% (9)	60% (64)	0.754	0.84	0.29–2.44
** BMI (kg/m ^2^ ) **	29.01 ± 6.0	28.8 ± 7.0	29.0 ± 5.8	0.864	0.99	0.91–1.09
< 25	25% (31)	31% (5)	25% (26)	0.566	1.40	0.44–4.40
25–29.9	39% (47)	31% (5)	40% (42)	0.523	0.69	0.22–2.14
30–34.9	20% (25)	19% (3)	21% (22)	0.853	0.88	0.23–3.37
35–39.9	9% (11)	12% (2)	8% (9)	0.604	1.54	0.30–7.87
> 40	7% (8)	6% (1)	7% (7)	0.958	0.94	0.11–8.21
**Age (years)**	56.4 ± 12.9	58.1 ± 13.2	56.1 ± 12.9	0.572	1.01	0.97–1.06
< 60	56% (68)	50% (8)	57% (60)	0.621	0.77	0.27–2.20
60–79	40% (49)	38% (6)	41% (43)	0.816	0.88	0.30–2.60
≥ 80	4% (5)	12% (2)	3% (3)	0.096	4.90	0.75–31.96
**Dyslipidemia**	**15% (18)**	**38% (6)**	**11% (12)**	**0.010**	**4.70**	**1.45–15.25**
**Hypertension**	61% (75)	81% (13)	58% (62)	0.094	3.08	0.83–11.44
**Diabetes**	23% (28)	31% (5)	22% (23)	0.400	1.64	0.52–5.20
**Sedentarism**	**9% (11)**	**25% (4)**	**7% (7)**	**0.026**	**4.71**	**1.20–18.49**
**Smoking**	12% (15)	19% (3)	11% (12)	0.404	1.81	0.45–7.27
**METs (DASI)**	5.6 ± 1.2	5.1 ± 1.3	5.6 ± 1.2	0.141	0.69	0.43–1.13
< 4	10% (12)	**25% (4)**	**8% (8)**	**0.040**	**4.08**	**1.07–15.62**
4–6.9	76% (93)	69% (11)	77% (82)	0.453	0.64	0.20–2.04
> 7	14% (17)	6% (1)	15% (16)	0.358	0.38	0.05–3.04
**ASA**		**2.1 ± 0.5**	**1.8 ± 0.5**	**0.035**	**3.67**	**1.09–12.33**
1	18.0% (22)	6% (1)	20% (21)	0.217	0.27	0.03–2.16
2	75.4% (92)	75% (12)	75% (80)	0.967	0.97	0.29–3.29
3	6.6% (8)	19% (3)	5% (5)	0.051	4.66	1.0–21.82
4	0% (0)	0% (0)	0% (0)	−	−	−

**Abbreviations:** 95% CI, 95% confidence interval; ASA, American Society of Anesthesiologists' physical status classification system; BMI, Body Mass Index; DASI; Duke Activity Status Index; MET; metabolic equivalent of task; OR, odds ratio; SD, standard deviation; THA, total hip arthroplasty.

**Notes:** Dichotomous data are presented as a percentage of occurrence (number) and numerical data as mean ± SD. Comorbidities with a prevalence of less than 15% were not reported, and none were identified as a risk factor. *p* -value referring to the Wald test.

Other comorbidities observed were chronic kidney disease (5%), hypothyroidism (4%), venous insufficiency (2%), and asthma (2%).

The classification according to the evaluated risk scores is presented in Table 2 , except for ASA, which is evaluated as a risk factor and a potential component of the developed tool, as shown in Table 1 .

**Table 2:** Risk scores

RCRI		CRI-TJA		MPOG	
**1**	91.0% (111)	**0**	37.7% (46)	**0**	0% (0)
**2**	8.2% (10)	**1**	59.0% (72)	**1–5**	74.6% (91)
**4**	0.8% (1)	**2**	3.3% (4)	**6–10**	23.7% (29)
**5**	0% (0)	**3**	0% (0)	**11–15**	1.63% (2)
				**> 15**	0% (0)

**Abbreviations:** CRI-TJA, cardiac risk index for total joint arthroplasty; MPOG, multicenter perioperative outcomes group; RCRI, revised cardiac risk index.

**Note:** Dichotomous data are presented as a percentage of occurrence (number).

The developed model corresponds to the Logit equation (p) = -8.89 + oct*1.76 + disl *1.47 + hip*0.05 + sed*1.65 + DASI4*1.60 + ASA*1.02 , where *p* is the estimated probability of occurrence of adverse events, *oct* indicates whether the patient is an octogenarian, *disl* if dyslipidemic, *hip* if hypertensive, *sed* if sedentary, *DASI4* if less than 4 METs estimated by DASI, and *ASA* is the numerical value of this risk score.

The optimal threshold identified for the model was 0.20. The model performed better than ASA ( *p* = 0.002), RCRI ( *p* = 0.007), CRI ( *p* = 0.002), and MPOG ( *p* < 0.001), as shown in Fig. 1 .

**Fig. 1:**
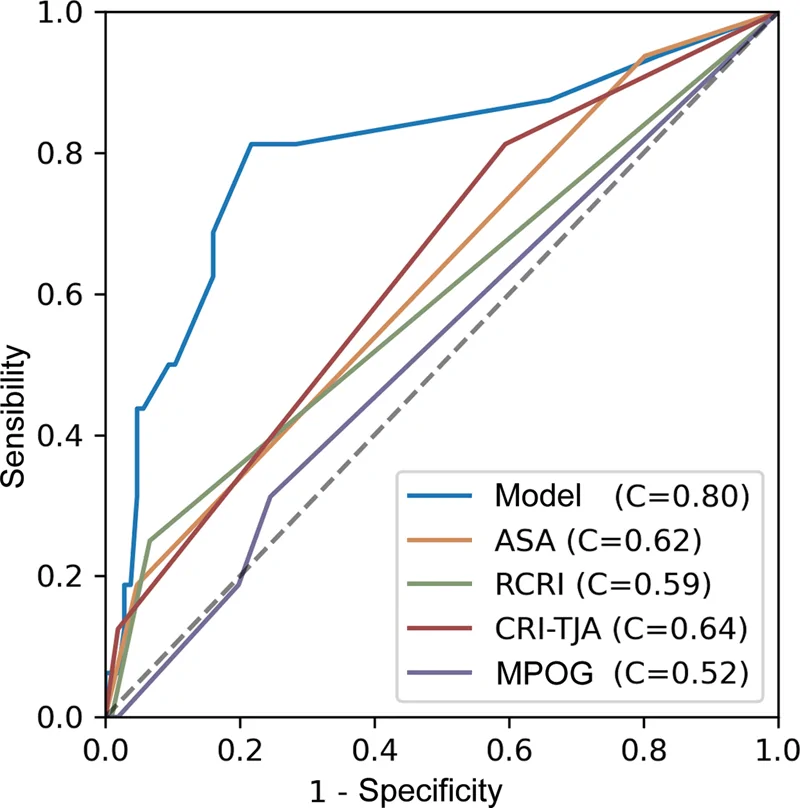
Performance of the developed model and the risk scores evaluated, presented by the ROC curve and quantified by the area under the ROC (C-statistic). **Abbreviations:** ASA, American Society of Anesthesiologists' physical status classification system; RCRI, revised cardiac risk index; CRI-TJA: cardiac risk index for total joint arthroplasty; MPOG, Multicenter Perioperative Outcomes Group; ROC, receiver operating characteristics.

To use the UPNEURO-INTO tool, the professional must access the link through a computer or mobile device and inform the patient's data. The DASI is presented in the left panel of the screen and can be completed either directly in the interview format or through prior registration. The result (expressed in METs) is presented in real time in the space below the questionnaire. In the upper-right panel of the screen, the clinical characteristics at the time of hospitalization must be filled in using the check boxes, and the ASA level must be indicated in the menu below it. The probability estimate of adverse events will be updated in real time during data entry and displayed in the indicator in the bottom-right panel.

## Discussion

The current study identified risk factors associated with complications or readmissions among patients undergoing elective primary THA. Among these factors, dyslipidemia, sedentary lifestyle, functional capacity below 4 METs estimated by DASI, and progressive ASA classification stand out. Based on these findings, it was possible to develop a tool to estimate the risk of adverse events, with performance superior to the other scores tested.

The sample had a lower-than-expected mean age and male predominance, in contrast to previous data indicating a greater number of women older than 65 years undergoing THA. 1 24 The high proportion of cases of secondary OA (25%), including etiologies such as trauma, osteonecrosis, and developmental dysplasia of the hip, may contribute to this distinct age and demographic profile. Considering that the prevalence of dysplastic diseases is higher among men younger than 50 years, 1 the high proportion of cases of secondary OA (25%) may explain this atypical profile.

About 75% of patients were overweight or obese, in line with the literature 25 and the recognition of overweight and obesity 26 as risk factors for OA. 1 The most frequent comorbidities were SAH, diabetes mellitus (DM), and dyslipidemia, with prevalences higher than those described in other studies. 14 These findings are compatible with the phenotype of OA associated with metabolic syndrome, 27 marked by low-grade chronic inflammation, although there is still no consensus on a causal relationship. 28

The high percentage of risk factors for cardiovascular disease, such as SAH, DM, dyslipidemia, smoking, and sedentary lifestyle, reinforces the importance of a careful preoperative evaluation.

Few orthopedic studies have evaluated functional capacity using the DASI, although this score is considered useful for individuals with limited mobility. 29 The mean value (5.5 METs) for functional capacity in the study is similar to that measured by cardiopulmonary exercise testing (CPET) in patients with hip OA, 30 which supports the potential use of DASI as a viable tool in this population.

Most patients had moderate functional capacity, and 10% had an estimated capacity of less than 4 METs, a value lower than that observed in other surgical populations, 23 which might be attributable to the limitations imposed by OA more than to a cardiopulmonary deficit.

Most patients were classified as low risk: ASA 2, RCRI 1 point, CRI-TJA indicating a minimum score for 40% of the sample, and MPOG classifying 75% as low risk. The choice of these indices for comparison with the study tool is due to the widespread use of ASA and RCRI, the specificity of CRI-TJA for THA, and the fact that MPOG is a risk score developed using data from the Brazilian population.

The rate of adverse events (13.1%) is compatible with the literature. 5 6 There were no deaths, which may indicate a high standard of care but may also be related to the small sample size. The most frequent complications were local infection and anemia requiring blood transfusion, followed by tachyarrhythmias, prosthetic dislocation, hypertensive crises, acute renal failure, suspected DVT, and COPD decompensation—well-documented events after THA. 16

The readmissions occurred at a rate similar to that reported by Roger et al. 31 and higher than Sørensen et al., 32 being mostly motivated by infection and suspicion of DVT, in agreement with previous findings. 16

Dyslipidemia was identified as one of the isolated risk factors for complications, an unprecedented finding in orthopedic studies, although metabolic syndrome has already been associated with complications and readmission within 30 days after THA and total knee arthroplasty. 33 The presence of SAH showed a tendency to triple the risk of adverse events or readmissions, similar to that observed by Waterman et al., 14 while this risk seems to increase fivefold in patients aged 80 years or older, although the sample size may have limited statistical significance.

A sedentary lifestyle and a functional capacity of less than 4 METs were associated with a more than fourfold increase in the risk of adverse events. Previous studies have suggested this relationship using subjective criteria, such as climbing stairs 17 34 or objective measures, such as oxygen consumption on CPET. 35 The DASI has already shown predictive value in noncardiac surgeries, 23 36 suggesting its application in preoperative evaluations. Even the self-declaration of a sedentary lifestyle proved to be useful as a risk indicator.

Identifying patients with a functional capacity of less than 4 METs allows us to outline strategies for increased surveillance and targeted interventions, such as exercise programs prior to surgery. A reduction in hospital stay has already been demonstrated with preoperative physical interventions, 37 and technological tools can facilitate adherence even at a distance. 38

The ASA classification also correlated with adverse events, corroborating previous studies reporting higher rates of complications in patients with ASA ≥ 3. 6

Based on the identified factors, a logistic regression model was proposed to estimate the risk of adverse events after THA. The simplicity of the data used makes the tool applicable in clinical practice. The developed model outperformed the traditional scores analyzed, demonstrating greater sensitivity and specificity in predicting complications or readmissions.

The present study has several limitations. First, DASI was applied via an interview, and cardiorespiratory capacity was not measured by CPET, another standard for this evaluation. Laboratory parameters such as hemoglobin and creatinine, which could represent additional risk factors, were also not included. Dyslipidemia was identified only by self-report or by statin use, without laboratory confirmation to detect unknown cases or to evaluate treatment control.

Analysis by specific type of complication (cardiac, noncardiac clinics—e.g. DVT, and acute renal failure—and surgical) was not possible due to the limited number of events and sample size. Similarly, the evaluation of myocardial injury would require tests such as an electrocardiogram and troponin dosage, which were not performed.

Additionally, as this is a pilot study, the prediction model has not yet undergone external validation, a step planned for later phases of the research.

On the other hand, the study has strengths: it is prospective, pioneering in the use of DASI in the preoperative period of THA, also proposing a specific risk prediction model for this population. The development of a computational prototype also highlights the proposal's clinical applications.

Despite the limitations, the results offer new perspectives on risk estimation in THA. Studies with larger sample sizes and across different contexts are needed to validate and improve the model. Future inclusion of frailty markers may enrich the analyses. 39

It is important to emphasize that objective risk estimates can guide shared doctor-patient decisions, surveillance strategies, and measures on modifiable factors. The estimate offered by the tool presented here is especially reliable in the hospital context in which it was developed and is particularly useful in specialized hospitals. While its use may support decision-making in general hospital settings, it is important to keep in mind that patient profiles may change, so the magnitude of risk estimates may be inaccurate.

## Conclusion

Dyslipidemia, sedentary lifestyle, reduced functional capacity (< 4 METs on DASI), and progressive ASA classification are isolated risk factors relevant to adverse events within 30 days after elective primary THA. Therefore, the need for an expanded preoperative evaluation is noteworthy, considering not only the ASA classification, but also functional aspects and comorbidities. The risk stratification tool proposed here demonstrated satisfactory accuracy, representing a potential resource for clinical practice. However, to implement the tool, external validation studies are necessary across different care contexts.
